# Home blood pressure monitoring for hypertension diagnosis by current recommendations: A long way to go

**DOI:** 10.1161/HYPERTENSIONAHA.121.18463

**Published:** 2021-12-02

**Authors:** Kelsey B. Bryant, Matthew B. Green, Daichi Shimbo, Joseph E. Schwartz, Ian M. Kronish, Yiyi Zhang, James P. Sheppard, Richard J. McManus, Andrew E. Moran, Brandon K. Bellows

**Affiliations:** 1 Division of General Medicine, Icahn School of Medicine at Mount Sinai, New York, NY, USA; 2 Division of General Medicine, Columbia Irving Medical Center, New York, NY, USA; 3 Stony Brook University, Stony Brook, NY, USA; 4 University of Oxford, Oxford, UK

## Introduction

Out-of-office blood pressure (BP) monitoring (e.g., home BP monitoring [HBPM] or ambulatory BP monitoring [ABPM]) to confirm a diagnosis of hypertension prior to treatment initiation after initial office screening is recommended by the United States Preventive Services Task Force (USPSTF) and 2017 American College of Cardiology and American Heart Association (ACC/AHA) BP guidelines.^1, 2^ One tool that may be used to help identify those in need of confirmatory BP monitoring is the PRedicting Out-of-OFfice BP (PROOF-BP) algorithm, which uses office BP measurements and clinical characteristics to predict a patient’s out-of-office BP.^3^ Though many providers report recommending out-of-office BP monitoring to patients, the baseline frequency of its use for specific indications, such as confirming a diagnosis of hypertension, is not known.^4^ Further, barriers relevant to the accessibility and affordability of out-of-office BP monitoring have led to concerns that there may be disparities in the uptake of hypertension screening recommendations.^5^ This analysis examined how historical use of HBPM aligns with current out-of-office BP monitoring recommendations for hypertensive US adults without a previous hypertension diagnosis, and how HBPM use varies by patient characteristics.

## Methods

Adults aged ≥20 years without a diagnosis of hypertension or antihypertensive medication use and a high office BP (≥130/80 mmHg) who participated in the National Health and Nutrition Examination Survey (NHANES) 2009-2014 cycles were identified (n= 7,185). Included participants had complete data needed to apply the ACC/AHA BP guideline criteria and PROOF-BP algorithm (i.e., age, sex, at least three office SBP and DBP readings, body mass index, and history of CVD).^3^ Participants who reported self-initiated or physician-recommended HBPM were categorized as having used or been told to use HBPM. Participants were categorized as having met criteria to undergo out-of-office BP monitoring according to the 2017 ACC/AHA recommendations if they had a mean office SBP/DBP 130-159/<100 mmHg and according to PROOF-BP if they had a predicted out-of-office BP 120-134/75-84 mmHg. The age-adjusted proportion of individuals that would meet criteria for out-of-office BP monitoring who reported using or being told to use HBPM was examined overall and was compared by race/ethnicity (i.e., non-Hispanic white, non-Hispanic Black, Hispanic, and other), sex, health insurance status, and source of routine health care. All analyses were performed using SAS (version 3.8; Cary, North Carolina) and were weighted to be representative of the 2013-2014 US adult population.

## Results

An estimated 31.4 million US adults did not have diagnosed hypertension, were not taking antihypertensive medications, and had an office BP ≥130/80 mmHg. Of the 95.3% (29.3 million) who would have met criteria to undergo out-of-office BP monitoring by the ACC/AHA guidelines, only 3.6% (1.1 million) were told to use HBPM, and 15.7% (4.7 million) had used HBPM (Figure). There were no differences in HBPM use by race/ethnicity, sex, health insurance status, or source of routine healthcare. Though the PROOF-BP algorithm would have recommended fewer individuals for out-of-office BP monitoring (61.9%, 19.5 million), the age-adjusted proportion who were told to use (2.6%, 0.5 million) or used (13.8%, 2.7 million) HBPM was similar overall, and differences were not identified by any of the patient characteristics examined.

## Discussion

There is a substantial gap between baseline out-of-office BP thresholds for recommended out-of-office BP monitoring and recent HBPM use and recommended use among US adults without a diagnosis of hypertension and a high office BP (≥130/80 mmHg). Among those with a high office BP who would now meet criteria for out-of-office BP monitoring according to ACC/AHA guidelines or the alternative PROOF-BP strategy, 24.9 million and 16.7 million, respectively, had not used HBPM to confirm hypertension. Evidence-based recommendations from the 2021 USPSTF report and 2017 ACC/AHA guideline stress the need for confirmatory out-of-office BP measurement prior to the initiation of antihypertensive medications. To meet these recommendations, HBPM use must dramatically increase, and will require removing systemic barriers to use, including insurance coverage for home BP devices and reimbursement for patient training in home BP use.^4^ The use of a telemonitoring system may improve ease of HBPM use for both physicians and patients, but also introduces additional cost and logistic considerations. Although we did not observe statistically significant differences by race/ethnicity or other patient characteristics, health systems should ensure that implementation plans are equitable and close rather than widen racial/ethnic and geographic hypertension disparities.^5^ These findings are limited in that NHANES had office BP from a single visit and were collected prior to recent screening recommendations; thus, our analyses do not reflect the impact of these guidelines on clinician or patient uptake of HBPM for hypertension diagnosis. Additionally, the impact of the coronavirus disease 2019 (COVID-19) pandemic, which accelerated adoption of remote patient management, on current HBPM use for hypertension diagnosis is unknown. Further, our analyses may underestimate out-of-office BP monitoring as ABPM data are not available in NHANES. However, these data do demonstrate the immense number of individuals who are eligible for out-of-office BP monitoring as part of an evidence-based screening algorithm and quantify an unmet opportunity for clinicians and health systems to improve the quality of hypertension screening.

## Figures and Tables

**Figure:**
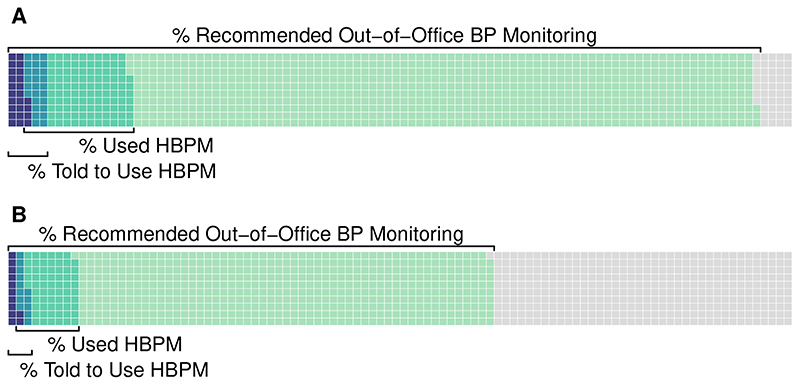
Out-of-office blood pressure monitoring recommendations and self-reported use and physician-recommended use of HBPM in NHANES 2009-2014. BP – blood pressure, HBPM – home blood pressure monitoring, NHANES – National Health and Nutrition Examination Survey Notes: The figure shows US adults (age ≥20 years) from the NHANES 2009-2014 cycles without diagnosed hypertension, not using antihypertensive medications, and an office blood pressure (BP) ≥130/80 mmHg (n=7,185). **Panel A** shows the proportion who would meet criteria to undergo out-of-office BP measurement by the 2017 American College of Cardiology and American Heart Association (ACC/AHA) BP guidelines and the age-adjusted proportion of those who either used or were told to use HBPM by a healthcare provider. **Panel B** shows the proportion who would meet criteria to undergo out-of-office BP measurement by the PRedicting Out-of-OFfice Blood Pressure (PROOF-BP) algorithm and the age-adjusted proportion of those who either used or were told to use HBPM by a healthcare provider.

**Table:** Characteristics and HBPM use among included participants from NHANES 2009-2014.

Characteristics	Overall	Age Group		
		20-44	45-64	65+
N	7,185	4,343	2,205	637
Female (%)	48.3%	47.6%	49.1%	51.0%
Race/Ethnicity (%)				
White (%)	68.4%	61.3%	77.2%	86.1%
Black (%)	8.8%	10.4%	7.1%	4.1%
Hispanic (%)	15.6%	19.4%	10.9%	5.9%
Other (%)	7.2%	8.9%	4.8%	3.9%
Source of usual care (%)	79.9%	75.2%	85.2%	94.5%
HBPM Use (%)	12.1%	10.1%	13.6%	22.0%

HBPM – home blood pressure monitoring, NHANES – National Health and Nutrition Examination Survey

Notes: Participants from the NHANES 2009-2014 cycles were included if they were adults (aged ≥20 years) without diagnosed hypertension, not using antihypertensive medications, and had an office blood pressure ≥130/80 mmHg. Included participants were required to have complete data on age, sex, at least three office blood pressure readings, body mass index, and history of cardiovascular disease.
